# Co-Infection of Oral *Candida albicans* and *Porphyromonas gingivalis* Is Associated with Active Periodontitis in Middle-Aged and Older Japanese People

**DOI:** 10.3390/medicina58060723

**Published:** 2022-05-28

**Authors:** Iori Oka, Hideo Shigeishi, Kouji Ohta

**Affiliations:** Department of Public Oral Health, Program of Oral Health Sciences, Graduate School of Biomedical and Health Sciences, Hiroshima University, Hiroshima 734-8553, Japan; m212911@hiroshima-u.ac.jp (I.O.); otkouji@hiroshima-u.ac.jp (K.O.)

**Keywords:** *Candida albicans*, *Porphyromonas gingivalis*, periodontitis, polymerase chain reaction

## Abstract

*Background and Objectives: Candida albicans* can be detected in subgingival sites of patients with periodontitis. However, the association between oral *Candida albicans* and periodontitis has not been fully elucidated in Japanese adults. The aim of this study is to clarify the relationship between oral *Candida albicans* infection/co-infection of oral *C. albicans* and *Porphyromonas gingivalis* and periodontitis among middle-aged and older Japanese people. *Materials and Methods:* Eighty-six patients (mean age 70.4 years) who visited the Hiroshima University Hospital from April to September 2021 were investigated in this study. Oral swab samples were collected from the tongue surface. *C. albicans* and *P. gingivalis* DNA was detected by real-time PCR using specific DNA primer sets. *C. albicans*-positive participants were classified into two groups according to the presence or absence of intron insertion of *C. albicans* DNA by PCR analysis. *Results: C. albicans* was detected in 22 (25.6%) of the 86 patients. Patients in their 80s recorded a higher *C. albicans*-positive rate (35.3%) compared with other participants. However, there was no significant association between the *C. albicans* positivity rate and clinical parameters such as sex, age, systemic disease, denture use, or oral health status. Of the 22 *C. albicans*-positive participants, 10 participants (45.5%) had *C. albicans* with intron insertion; 70% of participants who had *C. albicans* with intron insertion exhibited ≥6 mm probing depth. *C. albicans*/*P. gingivalis* co-infection was found in 12 patients (14%). Importantly, binomial logistic regression analysis revealed that *C. albicans*/*P. gingivalis* co-infection was significantly associated with ≥6 mm periodontal pockets with bleeding on probing (*p* = 0.02). *Conclusions:* Co-infection of *C. albicans* and *P. gingivalis* is involved in active periodontitis in middle-aged and older people.

## 1. Introduction

It is thought that the dysbiosis of the subgingival microbiome is importantly associated with the development of periodontitis [1]. *Treponema denticola*, *Tannerella* *forsythia*, and *Porphyromonas gingivalis*, the so-called red complex bacteria, are thought to be major periodontal pathogens [2]. Recent studies revealed that not only periodontopathic bacteria but also herpes viruses (e.g., herpes simplex virus 1, Epstein-Barr virus, and human cytomegalovirus) and oral *Candida albicans* are involved in periodontitis [3,4,5,6]. *C. albicans* can be detected in subgingival sites of patients with periodontitis, indicating that *C. albicans* has the ability to colonize inflammatory periodontal pockets and is importantly involved in periodontitis [7]. However, the relationship between oral *Candida albicans* and periodontitis has not been fully elucidated in Japanese adults. Additionally, the relationship between co-infection of oral *C. albicans* and periodontopathic bacteria such as *P. gingivalis* and periodontitis remains unknown.

Co-infection of periodontopathic bacteria and herpes viruses is importantly associated with active periodontitis [8]. In the interaction between periodontopathic bacteria and herpes virus, herpes virus-related local proinflammatory cytokines enhance the growth of periodontopathic bacteria [8]. In contrast, the Epstein–Barr virus can be reactivated by *P. gingivalis*-produced butyric acid [9]. Additionally, *P. gingivalis* facilitates the growth of *C. albicans* under aerobic and anaerobic conditions [10]. Therefore, it is vital to examine the co-existence of periodontopathic bacteria and other microorganisms for a better understanding of the pathogenesis of periodontitis. It is hypothesized that co-infection of periodontopathic bacteria and *C. albicans* is importantly involved in periodontal inflammation.

*C. albicans* can be classified into three genotypes in accordance with the presence or absence of transposable group I introns in the 25S rDNA gene [11,12]. The association between the genotype of oral *C. albicans* and the virulence activity (i.e., extracellular enzyme activity, adherence to epithelial cells, and biofilm formation) has been investigated [13,14]. However, no significant association between the genotype of oral *C. albicans* and fungal activity has been found [13,14]. The relationship between the genotype of *C. albicans* and periodontal health status in Japanese people remains unknown. The specific genotype of *C. albicans* may be associated with the severity of periodontal inflammation. The aim of this study was to clarify the relationship between the prevalence of oral *C. albicans* and periodontal health among middle-aged and older Japanese adults. In addition, the association between the *C. albicans* genotype and periodontal health status was investigated. Furthermore, the association of co-infection of oral *Candida albicans* and *P. gingivalis* and periodontal health status was examined. Recently, the periodontal inflamed surface area (PISA) has been used to quantify the total amount of inflamed periodontal tissue in the oral cavity [15]. The PISA value is a useful indicator to assess the severity of periodontitis. Therefore, PISA was employed to assess periodontitis in this study. The null hypothesis in this study was that there is no relationship between *C. albicans* and periodontitis.

## 2. Materials and Methods

### 2.1. Subjects

Eighty-six patients (mean age 70.4 years, range 43–86 years) who visited the Department of Oral Health of the Hiroshima University Hospital from April 2021 to September 2021 were included. No participants exhibited pseudomembrane formation or erythematous lesions in the oral cavity, which are features of oral candidiasis. This study has been approved by the Ethical Committee of Hiroshima University (No. E-1115, date of approval: 1 March 2018) and all participants signed a consent form.

### 2.2. Oral Sample Collection and DNA Extraction

Oral swab samples were collected from the tongue dorsum using a Orcellex^®^ Brush (Rovers Medical Devices, Oss, The Netherlands). The tongue dorsum was swabbed 10 times using a brush. A PureLink™ Microbiome DNA purification kit (Invitrogen; Thermo Fisher Scientific Inc., Waltham, MA, USA) was used for DNA extraction and purification.

### 2.3. Oral Investigation

Bleeding on probing (BOP) and probing depth were assessed at six sites (mesiobuccal, midbuccal, distobuccal, mesiolingual, midlingual, and distolingual) for each individual tooth. According to the community periodontal index for treatment needs (CPITN) [16], at least one ≥ 4 mm periodontal pocket indicates moderate periodontitis and at least one ≥ 6 mm periodontal pocket indicates severe periodontitis. Therefore, patients with ≥4 mm pockets and those with 6 mm pockets were investigated in this study. Then, the PISA and periodontal epithelial surface area (PESA) scores were calculated in accordance with the methods used in a previous study [15]. Next, plaque control record scores were recorded using a six-point method (mesiobuccal, midbuccal, distobuccal, mesiolingual, midlingual, and distolingual) using a plaque-disclosing solution to detect dental plaque accumulation. Systemic disease, denture use, and the number of remaining teeth were also recorded.

### 2.4. Detecting C. albicans DNA and P. gingivalis DNA by Real-Time PCR

Real-time polymerase chain reaction (PCR) analysis was carried out with a Thermal Cycler Dice^®^ Real Time System III (Takara, Osaka, Japan). Real-time PCR was performed using a THUNDERBIRD SYBR qPCR Mix (Toyobo Life Science, Osaka, Japan) in accordance with the manufacturer’s instructions. The amplification procedure was as follows: initial denaturation at 95 °C for 2 min, then 40 cycles of 95 °C for 1 min, 60 °C for 1 min, and 72 °C for 1 min. The following PCR primer sets were employed in this study: *C. albicans*, 5′-TTTATCAACTTGTCACACCAGA-3′ (sense), and 5′-ATCCCGCCTTACCACTACCG-3′ (antisense) [17]; and *P. gingivalis*, 5′-AGGCAGCTTGCCATACTGCG-3′ (sense) and 5′-ACTGTTAGCAACTACCGATGT-3′ (antisense) [18]. As described in our recent study [19], standard curves for 10-fold serial dilutions of *C. albicans* DNA were used to determine *C. albicans*-positive samples. Ten-fold serial dilutions of a *P. gingivalis* DNA positive sample (94.0 ng/μL–0.0094 ng/μL) were used to generate a standard curve (Supplementary Figure S1). Real-time PCR experiments were performed in duplicate.

### 2.5. PCR Analysis for Genotype Identification of C. albicans

PCR analysis was carried out using an Eppendorf Mastercycler EP Gradient S Thermal Cycler (Eppendorf, Hamburg, Germany). A GoTaq^®^ Green Master Mix (Promega, Madison, WI, USA) was used to amplify the PCR products in accordance with the manufacturer’s instructions. The amplification procedure was as follows: 95 °C for 2 min, followed by 40 cycles of 95 °C for 1 min, 60 °C for 1 min, and 72 °C for 1 min. PCR products were analyzed using 2% agarose gel electrophoresis with ethidium bromide and an ultraviolet transilluminator (FAS4; Nippon Genetics Co., Ltd., Tokyo, Japan). The Loading Quick 100 bp DNA Ladder (Toyobo, Osaka, Japan) was employed as a molecular size marker for gel electrophoresis. The following PCR primer sets were used: *C. albicans*-intron, 5′-ATAAGGGAAGTCGGCAAAATAGATCCGTAA-3′ (sense), and 5′-CCTTGGCTGTGGTTTCGCTAGATAGTAGAT-3′ (antisense) [12].

### 2.6. Statistical Analysis

SPSS software, version 24.0 (SPSS Inc., Chicago, IL, USA) was employed for statistical analysis. Fisher’s exact test or the χ^2^ test were employed to assess significant associations between clinical factors and *C. albicans* or *C. albicans/P. gingivalis*. The Mann–Whitney U test was employed to evaluate significant differences in age, remaining teeth, BOP (%), PISA value, and PESA value between *C. albicans*- or *C. albicans/P. gingivalis*-positive and negative cases. Logistic regression analysis was performed to adjust for the potential confounding effects in this study. Binomial logistic regression analysis with forced entry was performed to examine the relationship between independent variables and *C. albicans/P. gingivalis* as the dependent variable. Variables with a *p* value of less than 0.20 through univariate analysis were considered to be independent variables. Multicollinearity was evaluated using a variance inflation factor (VIF), and independent variables with a VIF of less than 2 were included in the logistic regression analysis. An adjusted odds ratio was obtained from logistic regression analysis. *p* Values of less than 0.05 were considered to be statistically significant.

## 3. Results

### 3.1. Relationship between Oral C. albicans and Clinical Parameters

Oral *C. albicans* positivity was determined in a total of 86 oral samples using real-time PCR. The *C. albicans*-positive rate was 26.1%. Table 1 summarizes the association between *C. albicans* and clinical factors. Participants in their 80s had a higher *C. albicans*-positive rate (35.3%) compared with other participants. There was no significant difference between the *C. albicans* positivity rate and clinical factors such as sex, age, systemic disease, or denture use. The association between oral *C. albicans* and oral health status was also investigated (Table 1). Participants with ≥6 mm periodontal pockets with BOP exhibited a higher *C. albicans*-positive rate (38.5%) compared with those without ≥6 mm periodontal pockets with BOP (23.3%). However, no significant association between *C. albicans* positivity and ≥6 mm periodontal pockets with BOP was found. *C. albicans*-positive participants recorded lower plaque control record scores compared with *C. albicans*-negative participants. No significant association was found between the *C. albicans* positivity rate and plaque control record scores.

### 3.2. Relationship between Intron Insertion of C. albicans and Clinical Parameters

**Figure 1:**
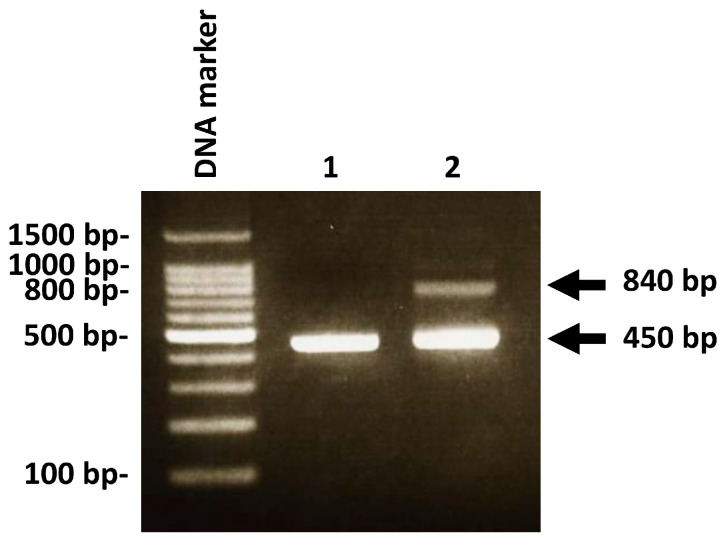
Polymerase chain reaction (PCR) detection of group I intron insertion in *C. albicans* positive cases. Lane 1: PCR products of 450 bp alone were found. Lane 2: PCR products of 450 bp and 840 bp were found. PCR products of 840 bp indicate the insertion of an intron into *C. albicans* DNA.

The *C. albicans* genotype can be classified into the following three types: genotype A (single band of 450 bp), genotype B (single band of 840 bp), and genotype C (two bands of 450 and 840 bp) in accordance with a previously reported study [11]. However, it was impossible to classify *C. albicans* into these three genotypes because DNA extracted from oral rinse samples was used for PCR analysis in this study. Therefore, *C. albicans*-positive participants were classified into two groups according to the presence or absence of a PCR band of 840 bp (i.e., group I intron insertion) (Figure 1).

Of the 22 *C. albicans*-positive participants, 12 participants (54.5%) had no *C. albicans* with intron insertion (Table 2). In contrast, 10 participants (45.5%) had *C. albicans* with intron insertion. No *C. albicans*-positive participants showed a single band of 840 bp alone. The mean age of participants who had *C. albicans* with intron insertion was greater than that of those who had *C. albicans* without an intron insertion. Participants who had *C. albicans* with an intron insertion exhibited higher plaque control record scores compared with those who had *C. albicans* without an intron insertion. However, no significant association was found between *C. albicans* with an intron insertion and plaque control record scores. Importantly, 70% of participants who had *C. albicans* with intron insertion exhibited ≥6 mm probing depth. In addition, participants who had *C. albicans* with intron insertion recorded higher PISA values than those without *C. albicans* with intron insertion. However, there was no significant association between intron insertion of *C. albicans* and PISA value in *C. albicans*-positive participants.

### 3.3. Relationship between Oral C. albicans/P. gingivalis and Clinical Parameters

The positive rate of co-infection of *C. albicans* and *P. gingivalis* was 14%. Table 3 summarizes the association between co-infection of *C. albicans* and *P. gingivalis* and clinical factors. Participants in their 80s showed a higher rate of co-infection of *C. albicans* and *P. gingivalis* (23.5%) compared with other participants. However, there was no significant difference between co-infection of *C. albicans* and *P. gingivalis* and clinical factors such as sex, age, or medical history. Denture use was significantly associated with co-infection of *C. albicans* and *P. gingivalis* (*p* = 0.02). Participants with ≥6 mm periodontal pockets with BOP had a higher *C. albicansP. gingivalis* positive rate (38.5%) compared with those without ≥6 mm periodontal pockets with BOP (9.6%). There was a significant association between co-infection of *C. albicans* and *P. gingivalis* and ≥6 mm periodontal pockets with BOP (*p* = 0.02). *C. albicans* and *P. gingivalis* double-positive participants exhibited higher PISA values than non-*C. albicans* and *P. gingivalis* double-positive participants, but no significant difference was found. *C. albicans* and *P. gingivalis* double-positive participants recorded lower plaque control record scores compared with non-*C. albicans* and *P. gingivalis* double-positive participants. No significant association was found between the *C. albicans* and *P. gingivalis* double-positive rate and plaque control record scores.

Next, binomial logistic regression analysis was conducted using co-infection of *C. albicans* and *P. gingivalis* as the dependent variable, and variables showing a *p* value of <0.20 such as remaining teeth, denture use, probing depth, and ≥6 mm periodontal pockets with BOP as independent variables. The Hosmer–Lemeshow test revealed a good fit of the model (*p* = 0.29). A significant association was found between co-infection of *C. albicans* and *P. gingivalis* and ≥6 mm periodontal pockets with BOP (*p* = 0.02) (Table 4).

## 4. Discussion

Although no significant association between *C. albicans* DNA and periodontal health status was found in this study, double infection of *C. albicans* and *P. gingivalis* was significantly associated with deep periodontal pockets with bleeding. Our results suggest that co-infection of *C. albicans* and *P. gingivalis* rather than *C. albicans* infection alone contributes to active periodontitis. *C. albicans* may be involved in periodontitis in cooperation with periodontopathic bacteria. The viability and hemagglutination activity of *P. gingivalis* was enhanced in the *P. gingivalis*/*C. albicans* mixed biofilm under low heme conditions, suggesting that *C. albicans* can enhance the virulence of *P. gingivalis* under the conditions of insufficient heme [20]. The capacity of *P. gingivalis* to invade gingival fibroblasts and epithelial cells was enhanced by mannans derived from *C. albicans* [21]. Therefore, *C. albicans* is thought to play a supportive role in the progression of periodontitis in the presence of *P. gingivalis*. Additionally, *C. albicans* may be associated with periodontopathic bacteria other than *P. gingivalis*. However, the relationship between *C. albicans* and periodontopathic bacteria other than *P. gingivalis* has not been investigated in this study. Possible associations between periodontopathic bacteria and the herpes virus have also not been elucidated in this study. Further additional study is necessary to clarify the relationship between periodontopathic bacteria and *C. albicans*/herpes virus in the pathogenesis of periodontitis.

Among the three different genotypes of *C. albicans*, genotype B (i.e., the genotype with a group I intron) was found to be most common in the oral cavity in both healthy participants and those with candidiasis [13]. Tantivitayakul et al. reported that *C. albicans* with intron insertion exhibited greater phospholipase activity and adherence capacity to epithelial cells compared with *C. albicans* without an intron insertion; however, there was no significant association between genotype and virulence characteristics such as phospholipase activity or adherence capacity in *C. albicans* [13]. In this study, participants who had *C. albicans* with intron insertion exhibited a greater surface area of periodontal inflammation than those without *C. albicans* with intron insertion. These results suggest that *C. albicans* with intron insertion may exhibit higher pathogenic potential. The specific genotype of *C. albicans* has high virulence activity and may be involved in severe periodontitis. However, it remains unknown which genotype of *C. albicans* is importantly associated with the severity of periodontitis. Additionally, the mean age was higher in *C. albicans*-positive participants who had *C. albicans* with intron insertion than in those who had *C. albicans* without an intron insertion. Increased infection of *C. albicans* with intron insertion may be associated with a decline in the immune response because of aging. However, it was impossible to determine the genotype of each *C. albicans* in this study. Further additional study will be required to identify the genotype of *C. albicans* using an isolated single colony of *C. albicans*.

Segata et al. divided the oral microbiome into three categories according to the location in the oral cavity: category 1 (microbiome of buccal mucosa, gingiva, and hard palate), category 2 (microbiome of saliva and tongue), and category 3 (microbiome of gingival plaque) [22]. The bacterial composition differed in each of the three categories, and bacterial genera associated with periodontitis were detected in the microbiome of the saliva and tongue [22]. It is thought that the microbiome of the saliva and tongue is importantly associated with the microbiome in the other groups and reflects the entire spectrum of the microbiome in the oral cavity. In addition, periodontopathic bacterial DNA can be detected by PCR using saliva and swabs from the tongue surface [23,24]. Therefore, it is thought that the sampling method (i.e., sample collection from the tongue surface) had little impact on the detection of periodontopathic bacteria such as *P. gingivalis* in this study.

The relationship between periodontopathic bacteria and PISA values was previously investigated in participants after adjustment for their potential confounding factors using propensity scores [25]. Participants with co-infection of *Tannerella* *forsythia* and *Treponema denticola* exhibited significantly higher PISA values than those without such co-infection [25]. The results highlight the importance of co-infection with red complex bacteria in periodontal inflammation severity. In contrast, no significant association was found between *P. gingivalis* and PISA values in participants after adjustment for their potential confounding factors [25]. Thus, *P. gingivalis* may determine the severity of periodontitis in cooperation with other periodontopathic pathogens such as *C. albicans*.

## 5. Conclusions

Co-infection with *C. albicans* and *P. gingivalis* rather than *C. albicans* infection alone is involved in active periodontitis among middle-aged and older Japanese people.

## Figures and Tables

**Table 1:** Relationship between oral *C. albicans* and clinical factors.

Clinical Factor (*n*)	*C. albicans*		*p*-Value
	(−) (*n* = 64)	(+) (*n* = 22)	
Age	69.7 ± 10.3	72.3 ± 9.4	0.43
Age in years			
40–49 (4)	4 (100%)	0 (0%)	0.64
50–59 (8)	6 (75%)	2 (25%)	
60–69 (25)	18 (72%)	7 (28%)	
70–79 (32)	25 (78.1%)	7 (21.9%)	
80–89 (17)	11 (64.7%)	6 (35.3%)	
Gender			
Male (25)	15 (60%)	10 (40%)	0.06
Female (61)	49 (80.3%)	12 (19.7%)	
Hypertension			
No (69)	53 (76.8%)	16 (23.2%)	0.36
Yes (17)	11 (64.7%)	6 (35.3%)	
Diabetes			
No (76)	56 (73.7%)	20 (26.3%)	1.0
Yes (10)	8 (80%)	2 (20%)	
Dyslipidemia			
No (72)	54 (75%)	18 (25%)	0.75
Yes (14)	10 (71.4%)	4 (28.6%)	
Remaining teeth	23.3 ± 5.8	21.9 ± 5.2	0.10
Denture use			
Non-user (55)	44 (80%)	11 (20%)	0.13
User (31)	20 (64.5%)	11 (35.5%)	
Plaque Control Record scores (%)	20.3 ± 15.9	16.4 ± 9.1	0.49
Probing depth			
<4 mm (18)	13 (72.2%)	5 (27.8%)	0.13
≥4 mm and <6 mm (42)	35 (83.3%)	7 (16.7%)	
≥6 mm (26)	16 (61.5%)	10 (38.5%)	
Bleeding on probing (%)	8.0 ± 8.9	8.4 ± 9.4	0.92
≥4 mm periodontal pocket with BOP			
No (48)	34 (70.8%)	14 (29.2%)	0.46
Yes (38)	30 (78.9%)	8 (21.1%)	
≥6 mm periodontal pocket with BOP			
No (73)	56 (76.7%)	17 (23.3%)	0.30
Yes (13)	8 (61.5%)	5 (38.5%)	
PISA (mm^2^)	93.6 ± 107.6	105.1 ± 143.9	0.66
PESA (mm^2^)	1023.0 ± 333.4	1007.5 ± 305.9	0.96

**Table 2:** Relationship between intron insertion of *C. albicans* and clinical factors in *C. albicans*-positive participants.

Clinical Factor (*n*)	*C. albicans* (+) (*n* = 22)		*p*-Value
	Intron Insertion (−) (*n* = 12)	Intron Insertion (+) (*n* = 10)	
Age	69.4 ± 9.8	75.8 ± 7.8	0.16
Age in years			
50–59 (2)	2 (100%)	0 (0%)	0.27
60–69 (7)	5 (71.4%)	2 (28.6%)	
70–79 (7)	3 (42.9%)	4 (57.1%)	
80–89 (6)	2 (33.4%)	4 (66.7%)	
Gender			
Male (10)	5 (50%)	5 (50%)	1.0
Female (12)	7 (58.3%)	5 (41.7%)	
Hypertension			
No (16)	9 (56.3%)	7 (43.8%)	1.0
Yes (6)	3 (50%)	3 (50%)	
Diabetes			
No (20)	11 (55%)	9 (45%)	1.0
Yes (2)	1 (50%)	1 (50%)	
Dyslipidemia			
No (18)	10 (55.6%)	8 (44.4%)	1.0
Yes (4)	2 (50%)	2 (50%)	
Remaining teeth	20.7 ± 6.5	23.4 ± 2.8	0.46
Denture use			
Non-user (11)	5 (45.6%)	6 (54.5%)	0.67
User (11)	7 (63.6%)	4 (36.4%)	
Plaque Control Record scores (%)	20.7 ± 6.5	23.4 ± 2.8	0.67
Probing depth			
<4 mm (5)	3 (60%)	2 (40%)	0.07
≥4 mm and <6 mm (7)	6 (85.7%)	1 (14.3%)	
≥6 mm (10)	3 (30%)	7 (70%)	
Bleeding on probing (%)	7.7 ± 10.1	9.2 ± 8.9	0.97
≥4 mm periodontal pocket with BOP			
No (14)	8 (57.2%)	6 (42.9%)	1.0
Yes (8)	4 (50%)	4 (50%)	
≥6 mm periodontal pocket with BOP			
No (17)	10 (58.8%)	7 (41.2%)	0.62
Yes (5)	2 (40%)	3 (60%)	
PISA (mm^2^)	80.1 ± 146.2	135.0 ± 142.7	0.58
PESA (mm^2^)	940.1 ± 342.1	1088.3 ± 1019.0	0.50

**Table 3:** Relationship between oral *C. albicans*/*P. gingivalis* and clinical factors.

Clinical Factor (*n*)	*C. albicans*/*P. gingivalis*		*p*-Value
	(−) (*n* = 74)	(+) (*n* = 12)	
Age	69.9 ± 10.0	73.1 ± 10.0	0.35
Age in years			
40–49 (4)	4 (100%)	0 (0%)	0.63
50–59 (8)	7 (87.5%)	1 (12.5%)	
60–69 (25)	21 (84%)	4 (16%)	
70–79 (32)	29 (90.6%)	3 (9.4%)	
80–89 (17)	13 (76.5%)	4 (23.5%)	
Gender			
Male (25)	20 (80%)	5 (20%)	0.32
Female (61)	54 (88.5%)	7 (11.5%)	
Hypertension			
No (69)	59 (85.5%)	10 (14.5%)	1.0
Yes (17)	15 (88.2%)	2 (11.8%)	
Diabetes			
No (76)	65 (85.5%)	11 (14.5%)	1.0
Yes (10)	9 (90%)	1 (10%)	
Dyslipidemia			
No (72)	60 (83.3%)	12 (16.7%)	0.20
Yes (14)	14 (100%)	0 (0%)	
Remaining teeth	23.3 ± 5.6	21.15 ± 6.3	0.16
Denture use			
Non-user (55)	51 (92.7%)	4 (7.3%)	0.02
User (31)	23 (74.2%)	8 (25.8%)	
Plaque Control Record scores (%)	19.6 ± 15.0	17.0 ± 10.7	0.65
Probing depth			
<4 mm (18)	14 (77.8%)	4 (22.2%)	0.06
≥4 mm and <6 mm (42)	40 (95.2%)	2 (4.8%)	
≥6 mm (26)	20 (76.9%)	6 (23.1%)	
Bleeding on probing (%)	7.7 ± 8.6	10.3 ± 11.2	0.52
≥4 mm periodontal pocket with BOP			
No (48)	42 (87.5%)	6 (12.5%)	0.76
Yes (38)	32 (84.2%)	6 (15.8%)	
≥6 mm periodontal pocket with BOP			
No (73)	66 (90.4%)	7 (9.6%)	0.02
Yes (13)	8 (61.5%)	5 (38.5%)	
PISA (mm^2^)	89.3 ± 106.9	141.5 ± 166.5	0.50
PESA (mm^2^)	1026.4 ± 318.2	973.2 ± 375.7	0.89

*p*-Values < 0.05 were considered statistically significant.

**Table 4:** Logistic regression analysis with *C. albicans*/*P. gingivalis* as dependent variable.

Clinical Variables	Adjusted Odds Ratio	95% Confidence Interval	*p*-Value
≥6 mm periodontal pocket with BOP	17.13	1.60–183.84	0.02

*p*-Values < 0.05 were considered statistically significant.

## Data Availability

All data generated or analyzed in this study are included in this article.

## Supplementary Materials

The following supporting information can be downloaded at https://www.mdpi.com/article/10.3390/medicina58060723/s1: Figure S1: Standard curve indicating a *Porphyromonas gingivalis* DNA positive sample vs. CT value.
