# Supplementary figures and images for: Co-Infection of Oral Candida albicans and Porphyromonas gingivalis Is Associated with Active Periodontitis in Middle-Aged and Older Japanese People
Source: Medicina (Kaunas). 2022 May 28;58(6):723. doi: 10.3390/medicina58060723 (PMC9227322; doi:10.3390/medicina58060723)

Supplementary figure S1

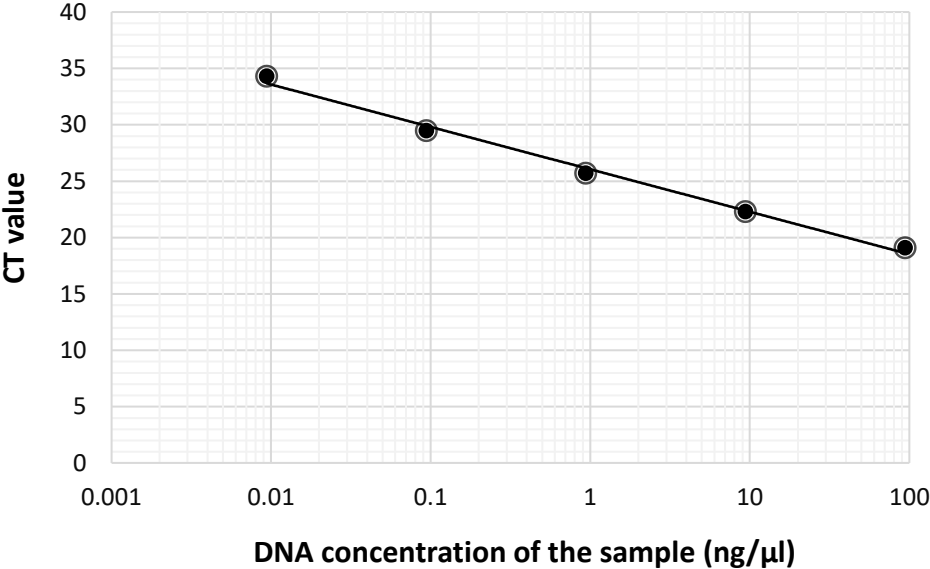

Supplement: Supplementary file 1 [file medicina-58-00723-s001.zip › medicina-1743745-supplementary.pdf]
